# Supplementary material for: Metal Oxide Nanostructures Enhanced Microfluidic Platform for Efficient and Sensitive Immunofluorescence Detection of Dengue Virus
Source: Nanomaterials (Basel). 2023 Oct 27;13(21):2846. doi: 10.3390/nano13212846 (PMC10648689; doi:10.3390/nano13212846)
Supplement: Supplementary file 1 [file nanomaterials-13-02846-s001.zip › nanomaterials-2646360-supplementary.pdf]

nanomaterials

Supplementary Materials

Metal Oxide Nanostructures Enhanced Microfluidic Platform for Efficient and Sensitive Immunofluorescence Detection of Dengue Virus

Pareesa Pormrungruang[1], Supranee Phanthanawiboon[2], Sukittaya Jessadaluk[1], Preeda Larpthavee[1], Jiraphon Tha-osing[2], Adirek Rangkasikorn[1], Navaphun Kayunkid[1], Uraiwan Waiwijit[3], Mati Horprathum[3], Annop Klamchuen[4], Tanapan Pruksamas[5], Chunya Puttikhunt[5], Takao Yasui[6], Mitra Djamal[7], Sakon Rahong[1*], and Jiti Nukeaw[1]

[1] College of Materials Innovation and Technology, King Mongkut's Institute of Technology Ladkrabang, Chalongkrung Rd., Ladkrabang, Bangkok 10520, Thailand
[2] Department of Microbiology, Faculty of Medicine, Khon Kaen University, Khon Kaen 40002, Thailand
[3] National Electronics and Computer Technology Center, National Science and Development Agency, Pathumtani 12120, Thailand
[4] National Nanotechnology Center, National Science and Development Agency, Pathumtani 12120, Thailand
[5] National Center for Genetic and Engineering and Biotechnology (BIOTEC), National Science and Development Agency, Pathumtani 12120, Thailand
[6] Department of Life Science and Technology, Tokyo Institute of Technology, B2-521, 4259 Nagatsuta-cho, Midori-ku, Yokohama Kanagawa 226-8501, Japan
[7] Department of Physics, Faculty of Mathematics and Natural Sciences, Bandung Institute of Technology, Bandung 46132, Indonesia

* Correspondence: sakon.ra@kmitl.ac.th

The optical density (OD) value of the anti-dengue envelope protein captured ELISA was measured at 450 nm. Non-diluted dengue serotype 3 (DENV-3) down to 10^{-7} dilution, along with a negative media control were applied. The positive result is the OD value above 0.088, the cut-off value calculated from the mean negative OD value within 3 standard deviation of the mean (±3SD).

Table S1. Capture ELISA result (n=3).

Virus	Non-diluted DENV-3	10^{-1}	10^{-2}	10^{-3}	10^{-4}	10^{-5}	10^{-6}	10^{-7}	Negative control
Mean OD	0.691	0.115	0.095	0.092	0.089	0.086	0.084	0.081	0.079

Mean negative = 0.079, Cut-off value = 0.088.
